# Evaluation of a point-of-care rapid HIV antibody test with insights into acute HIV symptomatology in a population with low prevalence

**DOI:** 10.1128/jcm.00620-24

**Published:** 2024-08-16

**Authors:** Tina I. Bui, Christopher W. Farnsworth, Neil W. Anderson

**Affiliations:** 1 Department of Pathology & Immunology, Washington University School of Medicine in St. Louis, Saint Louis, Missouri, USA; 2 Department of Pathology, University Hospitals Health System, Cleveland, Ohio, USA; Mayo Clinic Minnesota, Rochester, Minnesota, USA

**Keywords:** acute HIV, rapid testing, point-of-care, HIV

## Abstract

Many emergency departments (ED) use rapid human immunodeficiency virus (HIV) antibody tests as screening tools, despite limited sensitivity for detecting acute HIV infections. In a 4-year retrospective analysis of 1,192 patients, we evaluated the performance of a third-generation rapid HIV antibody assay tested at point-of-care (POC, Chembio Sure Check HIV 1/2) against in-lab fourth-generation screening (Abbott Architect Ag/Ab Combo). Compared to complete algorithmic testing, the POC test demonstrated a 92.5% sensitivity (95% CI = 84.6–96.5), 98.1% specificity (95% CI = 97.1–98.8), 99.5% negative predictive value (NPV; 95% CI = 98.8–99.8), and a 77.9% positive predictive value (PPV; 95% CI = 68.6–85.1). Notably, the POC test failed to detect 100% (3/3) of acute HIV infections (defined as Fiebig stage 2) and 3.8% (2/52) established HIV infections, where viral loads were 5.9, 6.7, and >7 log_10_ copies/mL. Symptoms such as fever, nausea/vomiting, malaise, headache, and photophobia were significantly associated with acute HIV infections diagnosed in the ED. The rapid HIV antibody test demonstrated high sensitivity, specificity, and NPV in our study population, reaffirming its effectiveness as a valuable screening tool. However, the low PPV and 100% failure to detect acute HIV infections underscore the importance of prioritizing in-lab fourth-generation HIV antigen/antibody combination immunoassays in cases of suspected acute HIV infection to ensure a timely and accurate diagnosis.

## INTRODUCTION

In the United States, a considerable proportion of individuals living with human immunodeficiency virus (HIV) remain unaware of their infection, with the Centers for Disease Control (CDC) estimates suggesting that one in eight people with HIV were undiagnosed between 2017 and 2021 (1). Rapid HIV tests have emerged as pivotal tools in HIV prevention efforts, facilitating faster detection of HIV infections, often within 30 minutes, while providing a user-friendly approach suitable for outreach, point-of-care (POC), and nonclinical settings. Since 2002, the Food and Drug Administration (FDA) has approved six rapid HIV antibody tests for use in the United States. In 2013, the FDA approved the rapid fourth-generation HIV antibody and antigen combination immunoassay, followed by fifth-generation HIV antibody and antigen in 2015, which reports where antibody and antigen results separately (2). FDA-approved third-generation lateral flow-based rapid tests continue to be utilized in clinical settings. However, they only exhibit up to 40% sensitivity when screening for early HIV infections, indicating a potential for undiagnosed acute HIV infections (3, 4). The low sensitivity of third-generation rapid HIV tests is partly due to a lack of p24 antigen detection, as featured in fourth- and fifth-generation HIV immunoassays.

Patients with acute HIV infections pose a unique challenge for clinical diagnostics and public health screening when using rapid HIV antibody tests due to the immunological response during the initial stages of infection. Seroconversion as measured by these assays does not typically occur until 20–30 days post-infection, resulting in a 2- to 3-week period of increased risk of transmission but decreased likelihood of detection due to a delay in antibody production in spite of high viral loads (5). This period prior to seroconversion is also categorized as stages I–III under the Fiebig classification (6). As they progress through Fiebig stages I–III, patients become detectable for HIV RNA and p24 antigen but remain seronegative by immunoblot. Consequently, individuals undergoing rapid HIV antibody testing which only target IgG (first and second generation) or IgG/IgM (third generation) may exhibit false negatives during this period, as HIV antibodies have not yet reached detectable levels. Erroneously negative results during this period pose considerable risk for HIV transmission as patients have high viral loads and are often still engaged in high-risk behavior (7, 8). Erroneously positive results pose potential anxiety for patients who falsely test positive for HIV.

To this end, the CDC has preferentially recommended the use of in-lab fourth-generation HIV antigen and antibody combination assays, which detect IgG/IgM and p24 antigen, as they are more sensitive and maintain comparable specificity compared to other currently available rapid tests (9). In-lab fourth-generation screening assays have been shown to decrease the window period approximately by 5 days (5, 9, 10) and have ~87.8% sensitivity for detecting acute HIV infections (3, 11, 12). This raises questions regarding the utility of lateral flow-based rapid tests in clinical settings alongside fourth-generation algorithmic testing. Few studies have directly examined the accuracy of third-generation rapid HIV tests performed at POC in clinical settings since the implementation of the CDC’s fourth-generation screening algorithm in 2014 (9). These studies exclusively report on the performance of rapid tests with minimal insights into scenarios which warrant in-lab screening over rapid testing at the POC (13, 14). Thus, there is a need for further investigation to determine a balance between lateral flow-based rapid tests and the recommended testing algorithm for diagnosing HIV infections.

The goal of this study was to retrospectively evaluate the impact of POC rapid HIV testing on the diagnosis of HIV, in a population with low HIV prevalence. Through an analysis of results from POC and in-lab HIV tests, coupled with clinical data, we sought to gain insights into the performance of rapid HIV testing in our patient population. We further aimed to characterize the clinical findings associated with false- versus true-positive screening results, which may guide in the overall interpretation of results in a clinical context. Finally, we aimed to quantify the different screening approaches across multiple years of clinical practice at our institution.

## MATERIALS AND METHODS

### Study design

Epic SlicerDicer was used to query the number of patients who received point-of-care rapid HIV antibody testing (Chembio Sure Check HIV 1/2) and/or in-lab fourth-generation screening (Abbott Architect HIV Ag/Ab Combo assay) across inpatient, outpatient, and the ED at Barnes Jewish Hospital (Saint Louis, MO) between 1/1/2018 and 12/31/2021. At our institution, point-of-care rapid HIV antibody tests are provided by a state-funded program for use in the ED, labor and delivery, and Women’s Assessment Clinic (a combination of inpatient and outpatient services). Clinicians in these settings have the option to order either or both tests at their discretion. However, a reactive POC rapid test must be followed up with the fourth-generation testing algorithm per CDC guidelines.

**Fig 1:**
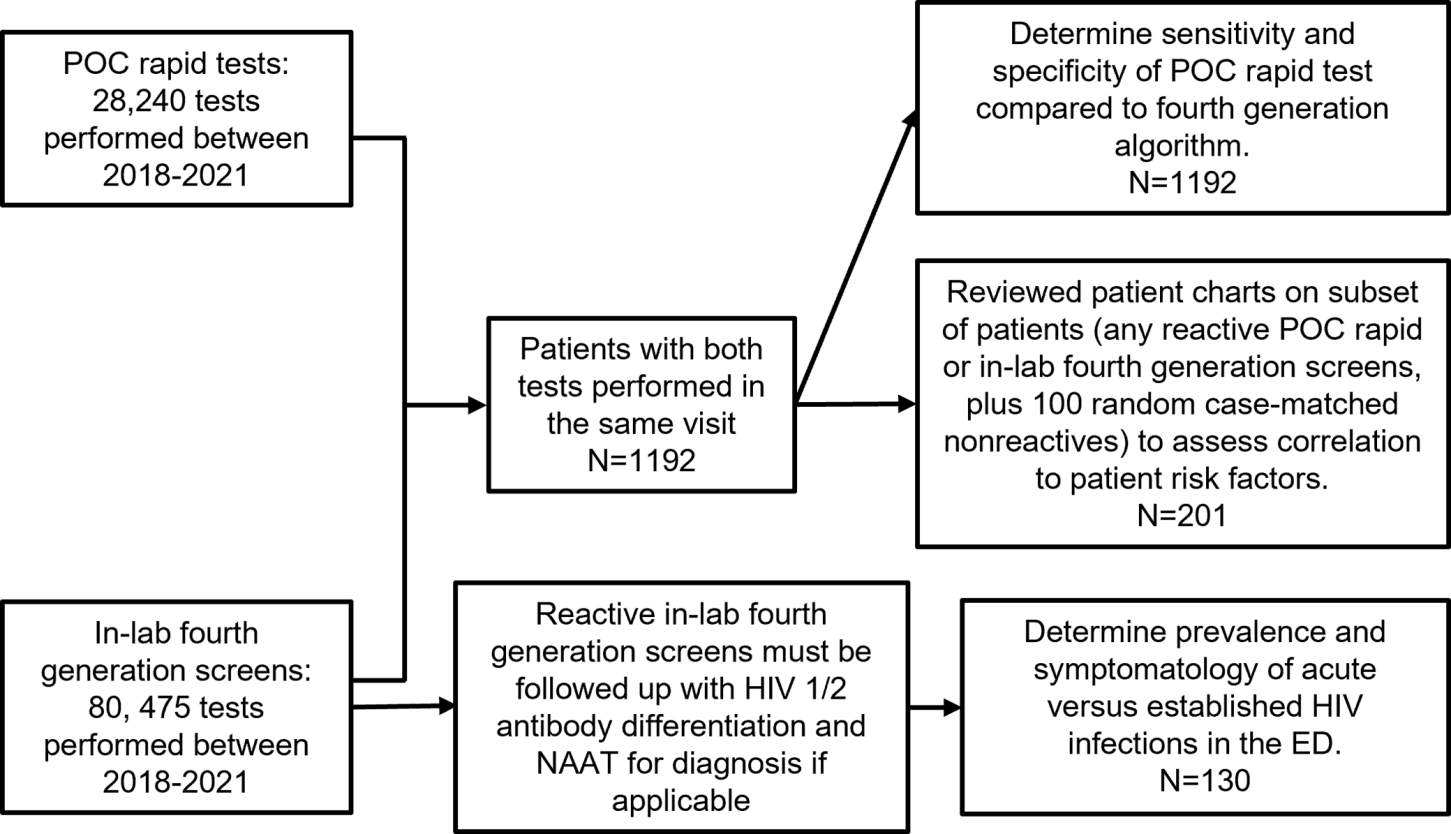
Identification of study population and subsets.

Figure 1 illustrates steps in the identification of the study population. Patients with both POC rapid test and in-lab fourth-generation screens performed for the same patient at the same visit within 7 days were selected (*N* = 1192). Patients with multiple paired testing at separate encounters were included in the study. This cohort (*N* = 1192) was used to determine the sensitivity and specificity of the POC rapid test compared to the fourth-generation testing algorithm to confirm HIV diagnosis. There were seven patients who had multiple POC rapid tests performed in a single encounter, results from the first POC test were included in the analysis for diagnostic performance.

We then performed a chart review for a subset of patients with reactive POC rapid test or in-lab fourth-generation screen, and an additional 100 patients case-matched by location and time with nonreactive test results (*N* = 201) were randomly selected to identify associations of patient demographics and risk factors to different testing patterns. The 201 patients included in the chart review were assessed for pregnancy status at time of testing and documented history of HIV infection with pre-exposure prophylaxis (PrEP) or current antiretroviral treatment (ART), intravenous drug use (IVDU), men who have sex with men status (MSM), other sexually transmitted infections (STIs) including hepatitis B, hepatitis C, chlamydia, gonorrhea, syphilis, and herpes simplex virus (HSV), and autoimmune disorders such as rheumatoid arthritis, lupus, multiple sclerosis, hemolytic anemia, and Sjogren’s syndrome.

Patients who were reactive on the in-lab fourth-generation screen during the study period were further staged based on results from the HIV ½ supplemental assay (Bio-Rad Geenius) and NAAT (cobas AmpliPrep/cobas TaqMan HIV-1 Test), as defined by Fiebig classifications (6). Acute HIV patients were defined as having reactive in-lab Architect HIV Ag/Ab Combo assay and HIV-1 NAAT, but nonreactive for HIV 1/2 antibodies by Bio-Rad Geenius HIV ½ (Fiebig stage 2). Established HIV patients tested reactive for both fourth-generation screen and Geenius HIV 1/2 antibody differentiation assays, plus detectable HIV-1 RNA (Fiebig stage 3).

### Symptomatology of patients with HIV infections presenting to ED

Based on the above definitions for staging of HIV disease, additional chart review was performed on a subset of patients with HIV that presented to the ED. Fever presentation included subjective fever reported by the patient or temperature measured >38°C in the ED.

### Statistical analysis

Fisher’s exact test was used to determine symptoms correlated with acute HIV infections and characteristics associated with different POC testing patterns. Multiple comparisons alpha value was corrected with Bonferroni’s correction. Unpaired Mann-Whitney *t*-tests were used to determine the significance between viral loads. All statistical analyses were performed using GraphPad Prism version 10.1.2.

## RESULTS

### Testing volume of lateral flow-based POC HIV antibody assay compared to in-lab fourth-generation screening assay

**Fig 2:**
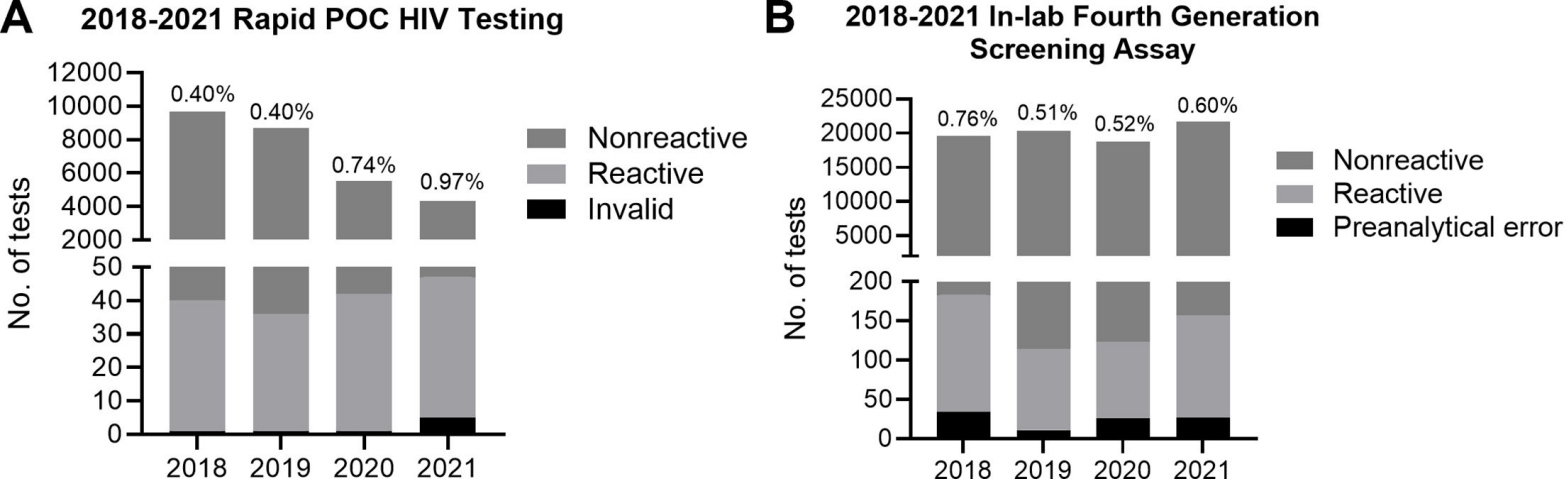
Testing volume and positivity rates of POC compared to in-lab fourth-generation HIV Ag/Ab screening assay. (**A**) Rapid POC HIV test results from Chembio Sure Check HIV 1/2 antibody test (total *n* = 28,240). (**B**) Results from in-lab fourth-generation testing *via* Abbott Architect Ag/Ab Combo assay (total *n* = 80,475). Percentages above each column represent annual positivity rates.

During the 4-year study period, a total of 28,240 rapid third-generation POC tests and 80,475 fourth-generation in-lab tests were performed across inpatient, outpatient, and ED settings. The positivity rate was <1% for both POC and in-lab testing with an overall average of 0.63% (yearly range, 0.40%–0.97%) for POC testing and 0.60% (range, 0.51%–0.76%) for in-lab fourth-generation assay (Fig. 2). There was a total of 157 reactive POC antibody tests from 151 unique patients. Of the 151 patients with reactive POC tests, 63.6% (96/151) were confirmed with in-lab fourth-generation screening within 7 days and 36.5% (35/96) of these patients were discharged before in-lab testing result. POC facilitated the diagnosis of 62 new HIV infections confirmed with fourth-generation algorithm testing. Among those with reactive POC tests who did not receive in-lab testing, 83.6% (46/55) had a past medical history of HIV diagnosis, 7.2% (4/55) had confirmatory testing at another facility after the POC test, 3.6% (2/55) went directly to NAAT, 1.8% (1/55) refused further workup, and 3.6% (2/55) were lost during linkage to confirmatory testing.

### Overall performance of POC HIV antibody assay

A total of 1,192 patients with paired POC and in-lab fourth-generation algorithm testing were identified. The performance of the POC was compared to complete fourth-generation algorithm testing as the gold standard for confirmed HIV diagnoses. The POC test exhibited a sensitivity of 92.5% (95% CI = 84.6–96.5) and a specificity of 98.1% (95% CI = 97.1–99.8). POC testing demonstrated a negative predictive value (NPV) of 99.5% (95% CI = 98.8–99.8) and a positive predictive value (PPV) of 77.9% (95% CI = 68.6–85.1). Chart review was performed on all patients with reactive POC tests or reactive in-lab fourth-generation screening assays, including a subset of patients (*n* = 100) who tested as nonreactive for both assays (Table 1). Younger age was associated with false-negative POC tests, where the median age was 23.5 y (IQR = 21.5–24.0) compared to medians of >30 y in other groups. MSM status, IVDU, and male sex were significant risk factors associated with confirmed HIV diagnoses. Other STIs and autoimmune disorders did not correlate with the different testing patterns. The POC assay demonstrated a false-negative rate of 0.50% (6/1,192) and a false-positive rate of 1.8% (21/1,192) during the 4-year period.

**TABLE 1:** Characteristics of patients with paired POC HIV antibody and in-lab fourth-generation algorithmic testing

POC HIV antibody result Confirmed HIV diagnosis by fourth-generation algorithm	Nonreactive Negative	Reactive Positive	Reactive Negative	Nonreactive Positive	
					*P*-value^*c*^
	*N* = 100	*N* = 74	*N* = 21	*N* = 6	
Age (IQR), year	38.5 (29–55.3)	33 (27–46)	32 (17–45)	23.5 (21.5–24)	**0.0162**
<18	7 (7)	0 (0)	6 (29)	0 (0)	**0.0003**
≥18	93 (93)	74 (100)	15 (71)	6 (100)	
Sex at birth					**<0.0001**
Female	53 (53)	17 (23)	14 (67)	1 (17)	
Male	47 (47)	57 (77)	7 (33)	5 (83)	
Race					0.0546
Asian	0 (0)	0 (0)	0 (0)	0 (0)	
Black	61 (61)	61 (82)	16 (76)	4 (67)	
White	34 (34)	10 (14)	5 (24)	2 (33)	
Other/unknown	5 (5)	3 (4)	0 (0)	0 (0)	
Pregnant	20 (38)	0 (0)	1 (7)	1 (100)	**0.0004**
IVDU	13 (13)	13 (18)	3 (14)	0 (0)	0.7395
Homeless	22 (22)	18 (24)	4 (19)	0 (0)	0.7003
PMH of HIV	0 (0)	12 (16)	0 (0)	1 (17)	**<0.0001**
HIV ART	0 (0)	3 (4)	0 (0)	0 (0)	
HIV PrEP	0 (0)	1 (1)	0 (0)	0 (0)	
MSM	0 (0)	22 (39)	1 (7)	4 (80)	**<0.0001**
Other STIs^*a*^	29 (29)	20 (27)	4 (19)	2 (33)	0.0825
Autoimmune disorders^*b*^	4 (4)	1 (1)	0 (0)	0 (0)	0.7086

^*a*^ Other STIs include hepatitis B, hepatitis C, chlamydia, gonorrhea, syphilis, and herpes simplex virus.

^*b*^ Autoimmune disorders include rheumatoid arthritis, lupus, multiple sclerosis, hemolytic anemia, and Sjogren’s syndrome.

^*c*^ Statistically significant results as determined by Fisher’s exact test are bolded.

Both POC rapid and in-lab fourth-generation testing are available 24/7 to providers and are ordered at their discretion at our institution. In-lab fourth-generation screening assays are immediately tested upon receipt in the clinical laboratory. Given this, we noted that 71.6% (144/201) patients had in-lab fourth-generation screens ordered after POC rapid result. Of those 57 who had in-lab fourth-generation screen ordered before the POC rapid test, 78.9% (45/57) were pregnant, minors, or had risk factors including MSM status, IVDU, homelessness, or previous STI diagnosis. The time to results may be impacted by provider ordering behavior. The median turnaround time (TAT) from POC test to reactive Geenius HIV ½ differentiation assay or positive NAAT (whichever came first) for true positives was 5.8 h (IQR = 3.6–8.9) and 25.1 h (IQR = 10.2–34.8) for false negatives. The median TAT for false positives from the POC test to the in-lab fourth-generation screen was 2.1 h (IQR = 1.4–2.6). Geenius HIV ½ antibody differentiation was only performed in 13/21 false positives, which were all negative.

### Performance of POC HIV antibody assay for detecting acute HIV infections

We identified a total of 208 patients with confirmed HIV diagnoses, of which 10 patients had acute HIV infections and 198 patients with established HIV infections. Of the 208 patients, POC HIV antibody testing was only performed in 26.4% (55/208) patients (Table 2). Among the 198 patients with established HIV infections, 12/198 had viral loads <20 copies/mL and 2/198 had viral loads > 10,000,000. By contrast, 5/10 acute HIV patients had viral loads > 10,000,000 copies/mL. Excluding these out-of-range RNA titers, the median log_10_ viral load for acute infections was 5.9 (95% CI = 5.8–6.7) compared to a median of 4.5 (95% CI = 4.4–4.7) in established infections. Notably, 100% (3/3) of POC tests failed to detect acute HIV infections, where viral loads were 5.9, 6.7, and >7 log_10_ copies/mL. These three patients with acute HIV infections were not on PrEP at the time of POC rapid testing. There were also two false negatives from patients with established HIV infections. These patients had no recorded history of antiretroviral therapy and exhibited viral loads of 4.5 and 4.9 log_10_ copies/mL.

**TABLE 2:** POC testing in acute versus established HIV infections^*a*^

Stage of disease based on testing^*b*^	In-lab fourth-generation HIV 1/2 Ab + p24 Ag	HIV 1/2 Ab differentiation^*c*^	NAAT^*c*^	POC test^*c*^	N
Acute HIV	Reactive	Nonreactive	Detected	Reactive	0
Acute HIV	Reactive	Nonreactive	Detected	Nonreactive	3
Established HIV	Reactive	Reactive	Detected	Reactive	50
Established HIV	Reactive	Reactive	Detected	Nonreactive	2^*d*^

^*a*^ HIV = human immunodeficiency virus, Ab = antibody, Ag = antigen, NAAT = nucleic acid amplification test. 211 persons were excluded from table due to lack of NAAT results or had undetectable viral loads.

^*b*^ Staging of disease is based on Fiebig classification of acute HIV infections.

^*c*^ Testing was performed within 7 days of in-lab fourth-generation screen.

^*d*^ A total of three patients were identified but one was excluded since NAAT was performed >7 days.

### Symptomatology of acute HIV versus established HIV infections in the ED

Among the 10 acute HIV cases, 7/10 were patients encountered in the ED and all three false-negative POC results in acute HIV cases were performed in the ED. Symptoms were assessed for patients presenting to the ED with acute HIV (*n* = 7) compared to established HIV infections (*n* = 123) in Table 3. The remaining 78 patients with HIV infections were encountered in other inpatient or outpatient settings. In those with acute HIV infections, fever, malaise, nausea/vomiting, and headache were among the most common symptoms (presented in >50% of patients). Photophobia was also reported more often in acute HIV patients than in those with established HIV (*P* < 0.0025). Weight loss, chills, rash, lymphadenopathy, arthralgia, and genital/rectal lesions were among the least documented in acute HIV patients. A significant proportion of acute HIV patients (71%) presented with fever in conjunction with three or more other symptoms compared to 7% in those with established HIV infections (*P* < 0.0001). In patients with established HIV infections, symptom presentation was extremely broad, with no one symptom documented in over 20% of patients.

**TABLE 3:** Symptoms of patients presenting with acute HIV vs established HIV infections in the emergency department^*a*^

Symptom	Acute HIV infection (*n* = 7)	Established HIV infection (*n* = 123)	*P-*value^*c*^
Fever^*b*^	5 (71)	21 (17)	**0.0036**
Weight loss	1 (14)	11 (9)	0.5009
Gastrointestinal			
Nausea/vomiting	5 (71)	21 (17)	**0.0036**
Diarrhea	2 (29)	16 (13)	0.2494
Abdominal pain	3 (43)	18 (15)	0.0831
Chills	1 (14)	20 (16)	>0.9999
Sweats	1 (14)	5 (4)	0.2874
Malaise	4 (57)	24 (20)	**0.0384**
Myalgia	2 (29)	9 (7)	0.1084
Headache	4 (57)	10 (8)	**0.0025**
Pharyngitis	0	18 (15)	0.5923
Rash	1 (14)	12 (10)	0.5305
Lymphadenopathy	1 (14)	1 (1)	0.1052
Arthralgia	1 (14)	2 (2)	0.1541
Oral lesions	0	7 (6)	>0.9999
Genital/rectal bleeding	0	1 (1)	>0.9999
Genital/rectal lesions	1 (14)	5 (4)	>0.9999
Photophobia	2 (29)	0	**0.0025**
Fever + ≥3 other symptoms	5 (71)	8 (7)	**<0.0001**
None of the above symptoms	0	28 (23)	0.3450
Unable to review symptoms	0	13 (11)	>0.9999

^*a*^ Of the 208 patients with HIV in Table 2, 78 were excluded in the above table because the patient encounter did not occur in the ED.

^*b*^ As reported by the patient or temperature >38°C.

^*c*^ Statistically significant results as determined by Fisher’s exact test are bolded.

## DISCUSSION

Rapid HIV antibody tests used at the POC are valuable tools for screening for HIV, particularly in high-risk populations. In this study, we found that the third-generation rapid test used at the POC facilitated the diagnosis of 62 new HIV infections that were confirmed with fourth-generation algorithm testing. However, acute HIV infection poses a unique challenge for clinical diagnostics and public health screening due to a window period where patients are viremic but seronegative. Notably, the POC test failed to detect 3/3 of acute HIV infections. Nonetheless, the third-generation rapid tests used at the POC demonstrated a high NPV of 99.5% and a PPV of 77.9%.

An important strength of this study was the retrospective assessment of results from a real-world setting where both POC and the gold standard fourth-generation algorithm testing were available to providers. Limited published studies have assessed the accuracy of lateral flow-based HIV tests used at the POC in untargeted populations. POC testing leverages non-laboratorian testing personnel and has been shown to suffer from higher error rates than in-lab testing (15). However, untargeted screening with POC tests funded by the state enables the testing of patients who might not otherwise receive HIV screening and offers an immediate opportunity for counseling. Previous studies have noted the high specificity and sensitivity of POC testing in high-risk populations (2, 16). Similarly in this study, the rapid antibody test used at the POC had high specificity and its utility as a screening assay was evident with an NPV of 99.5%, highlighting its effectiveness in accurately identifying individuals without HIV. However, a PPV of 77.9% emphasizes the need for caution and the availability of follow-up confirmatory testing. In our patient population, the POC antibody test demonstrated a 1.8% false-positive rate and 0.5% false-negative rate when compared to the gold standard in-lab fourth-generation screening assay. While this may have represented user error, it more likely reflects the lower diagnostic accuracy of third-generation rapid HIV testing.

In our study, only 3/10 acute HIV patients had a POC HIV antibody test performed, all of which failed to detect HIV infection. The poor performance of the POC test in detecting acute infections could be attributed to it being a third-generation assay, which only detects anti-HIV IgG and IgM, and therefore prone to missed diagnosis of acute HIV infections. Fourth-generation rapid tests, such as the Determine HIV-1 Ag/Ab Combo Rapid Test (RT), mitigate this issue by simultaneously detecting the p24 antigen and anti-HIV IgG/IgM. However, numerous studies have highlighted the suboptimal performance of the Determine Combo RT in identifying acute HIV infections when compared to laboratory-based fourth-generation assays such as the Architect Ag/Ab assay (3, 17–24). The Determine Combo RT has consistently reported a sensitivity range of 83%–88% in these studies with high-risk populations and as a screening assay (3, 12, 20). Thus, despite incorporating both antibody and antigen detection, fourth-generation rapid tests still exhibit lower sensitivity compared to their in-lab counterparts. Some argue that earlier recognition of acute symptoms and increased testing frequency could contribute to a reduction in HIV transmission from individuals in the acute phase (25). In this study, a large proportion (70%) of acute HIV patients were seen in the ED. Most patients with acute HIV infections present with nonspecific symptoms such as fever and malaise that are indicative of viral syndrome (26–28). In a multicenter study in the United States, untargeted screening in the ED resulted in 839 new HIV diagnoses, of which 122 or 14.5% were acute HIV infections (28). Similar to our study, patients with acute HIV infections commonly presented to the ED with symptoms such as fever, gastrointestinal issues (nausea/vomiting/abdominal pain), malaise, and chills (28). Importantly, photophobia and the presence of fever along with three other symptoms showed a strong association with acute HIV infections compared to other symptoms typically described. In instances where providers are given the choice to begin HIV testing with POC rapid or in-lab fourth-generation testing, these results promote prioritizing in-lab fourth-generation screening over POC tests for febrile patients with multiple symptoms of viral infection in the ED.

In this study, the median TAT for confirmatory results in cases of false negatives was 6.6 hours longer than false positives. False negatives may have been associated with recent infections characterized by undetectable antibody levels, which could have been more accurately identified with in-lab fourth-generation HIV Ag/Ab assays, potentially reducing the TAT. Viral suppression may result in undetectable antibody levels and lead to false negatives on POC rapid tests (29, 30), although the rate of PrEP and ART in our cohort was extremely low. Patients with false-negative POC tests were more likely to be younger adults, with no history of IVDU and therefore likely contracted HIV through sexual transmission. These findings underscore the need for confirmatory in-lab testing as misdiagnoses can cause patient anxiety (8) and contribute to the spread of HIV due to high viral loads in early infections (5). In our study, half of the acute HIV cases had viral loads of >10,000,000 copies/mL, indicating significantly higher infectivity. Given that undiagnosed individuals with HIV are estimated to contribute to up to 30% of new infections annually in the United States (31, 32), identifying instances when confirmatory testing is essential to accurately diagnose acute HIV infections may reduce HIV transmission.

During our 4-year retrospective review period, we report a positivity rate of <1% for both POC HIV antibody testing (Sure Check HIV 1/2) and in-lab fourth-generation screening (Architect HIV Ag/Ab Combo assay). Despite a decline in the volume of POC testing from 2018 to 2021, the POC test positivity rate increased from 0.40% to 0.97%. This change is likely attributed to a decline in the number of people seeking healthcare services at the ED during the COVID-19 pandemic without major changes to HIV incidence in the state of Missouri (33). By contrast, the volume of in-lab testing remained stable between 2018 and 2021, possibly due to the widespread ordering of in-lab tests across departments as opposed to POC testing, which is primarily offered in the ED.

Our study does have limitations. Despite this being a single-center study, we successfully mirrored national statistics, demonstrating that HIV disproportionately affects Black individuals and MSM (1). In our patient population, 81.3% (65/80) of confirmed HIV cases that underwent both POC and in-lab fourth-generation algorithm testing were Black. Yet, Black individuals only comprised 63.6% (758/1192) of the total screened population, indicating potential testing disparities. The low HIV prevalence in our population contributed to fewer observed cases of acute HIV infections and likely affected the false-positive and false-negative rates. During the study period, the prevalence of HIV in Saint Louis County was estimated to be 266 per 100,000 population compared to the national prevalence rate of 432.7 per 100,000 (1, 33). However, this also enhances the study’s generalizability, as many regions globally experience low HIV prevalence (34). Another limitation is the inability to analyze POC rapid testing by testing location due to inaccurate documentation of the testing site in our electronic medical record system, highlighting the need for change in recording POC rapid testing.

In conclusion, our findings support continued efforts for widespread HIV screening using third-generation rapid antibody tests used at the POC. However, POC rapid testing may lead to confusion if a laboratory can already deliver fourth-generation results in a relatively quick turnaround time. From specimen receipt to results, the median TAT time for in-lab fourth-generation screening in this study was 67 minutes (IQR = 57–85). Therefore, providers should prioritize more accurate in-lab fourth-generation testing for individuals presenting with multiple symptoms suggestive of viral syndrome to mitigate the risk of HIV transmission from undiagnosed acute cases. To this end, we have provided valuable insights into the merits and limitations of rapid HIV antibody screening within the current guidelines for fourth-generation algorithm testing.
